# Development of *Streptococcus equisimilis* Group G Mutant Strains with Ability to Produce Low Polydisperse and Low-Molecular-Weight Hyaluronic Acid

**DOI:** 10.52547/ibj.3789

**Published:** 2022-10-30

**Authors:** Bahareh Jafari, Malihe Keramati, Reza Ahangari Cohan, Seyed Mohammad Atyabi, Sara Ali Hosseinzadeh

**Affiliations:** 1 Department of Nano-Biotechnology, New Technologies Research Group, Pasteur Institute of Iran, Tehran, Iran;; 2 Department of Biological Sciences, Sciences and Research Branch, Islamic Azad University, Tehran, Iran

**Keywords:** Hyaluronic acid, Microbial production, Molecular weight, Mutation, * Streptococcus equisimilis *

## Abstract

**Background:**

Hyaluronic acid, a natural polymer with wide applications in biomedicine and cosmetics, is mainly produced by *Streptococcal* fermentation at industrial scale. In the present study, chemical random mutagenesis was used for development of *Streptococcus equisimilis* group G mutant strains with high HA productivity.

**Methods:**

The optimum of the pH of culture condition and cultivation time for HA production by wild strain group G were assessed. At first, two rounds of mutation at different concentrations of NTG was used for mutagenesis. Then, the nonhemolytic and hyaluronidase-negative mutants were screened on the blood and HA agar. HA productivity and molecular weight were determined by carbazole assay, agarose gel electrophoresis and specific staining. Moreover, stability of the high producer mutants was evaluated within 10 generations.

**Results:**

The results showed that the wild-type strain produced 1241 ± 2.1 µg/ml of HA at pH 5.5 and 4 hours of cultivation, while the screened mutants showed a 16.1-45.5% increase in HA production. Two mutant strains, named Gm2-120-21-3 (2470 ± 8.1 µg/ml) and Gm2-120-21-4 (2856 ± 4.2 µg/ml), indicated the highest titer and a consistent production. The Mw of HA for the mutants was less than 160 kDa, considering as a low Mw HA.

**Conclusion:**

The mutant strains producing a low polydisperse, as well as low Mw of HA with high titer might be regarded as potential industrial strains for HA production after further safety investigations.

## INTRODUCTION

Hyaluronic acid is a linear non-sulfated glycosaminoglycan comprising of GlcUA and N-acetylglucosamine moieties^[^^1^^,^^2^^]^. HA is found in mammalian tissues, *chloroviruses*^[^^3^^]^, *Streptococcus pyogenes* (group A), *S. zooepidemicus*, and* S. equisimilis* GCS and GGS^[^^4^^]^. Owing to viscoelasticity, non-immunogenicity, and biocompatibility, HA has a wide range of applications in biomedicine and cosmetics^[^^5^^]^. The biological function of HA depends on the Mw. Low Mw HAs (<1 megadalton) mediate leukocyte, fibroblast, and endothelial cell migration, as well as are involved in wound healing, whereas high Mw of HA mediate the hydration and lubrication functions^[^^6^^]^. 

Industrially, as HA extraction from rooster comb has been restricted due to safety concerns such as related avian allergens and viral contaminations, the fermentation of *Streptococcus *species, particularly *S. zooepidemicus* and GCS, is being employed^[^^4^^]^. For increasing the HA productivity, strain development or improvement, culture optimization, and purification adjustment have received lots of attention. The HA biosynthesis pathway of *Streptococcal* strains includes the polymerization of GlcUA-UDP and GlcNAc-UDP sugars by transmembrane HA synthase, which is encoded by *hasA* gene^[^^7^^]^. It has been reported that a strong *hasA* promoter often results in highly mucoid strains^[^^8^^]^. Single-point mutagenesis in the upstream region of *hasA* promoter would lead to an increase (5-16%) or a decrease (41%) in the HA productivity^[^^9^^]^, indicating the importance of genetic role in the HA production. Beside the genetic modifications, change in the medium composition and optimization of cultivation condition have demonstrated noticeable impacts on the production yield up to 6-7 mg/ml^[^^10^^,^^11^^]^. Moreover, the presence of streptolysin and hyaluronidase in the *Streptococcal* strains mediating subsequently the hemolysis and HA degradation, affects the polydispersity of final product^[^^12^^]^. 

In the present study, following the primary isolation of 10 nonpathogenic GCS and GGS^[^^13^^]^, a strain belongs to GGS was subjected to random chemical mutagenesis using NTG. The NTG mutagenesis mediates the alkylation of guanine and thymine, which in turn makes stable transition mutations between GC and AT in DNA strand^[^^14^^]^. Following random mutation, the negative-streptolysin and hyaluronidase-negative high-producing mutants were screened, and the consistency of morphology and HA production of high producers were assessed for 10 passages on a blood agar plate. Finally, Mw of the purified HAs was determined using agarose gel electrophoresis.

## MATERIALS AND METHODS

Strains and culture media

The *S. equisimilis* GGS-88 as wild strain (here after Gw) was subjected to random mutation and selection. This strain was selected based on the primary screening of 10 nonpathogenic GCS and GGS in the case of HA production^[^^13^^]^. All GCS and GGS strains were characterized by microbial and biochemical tests in accordance with our previous study^[^^15^^]^. *S. zooepidemicus *(ATCC no. of 35195) was used as the control strain in all experiments. Microorganisms were grown in BHI and blood agar (Difco, UK) for nonhemolytic (negative-streptolysin) selection.

Mutation and selection of GGS strains 

**Fig. 1:**
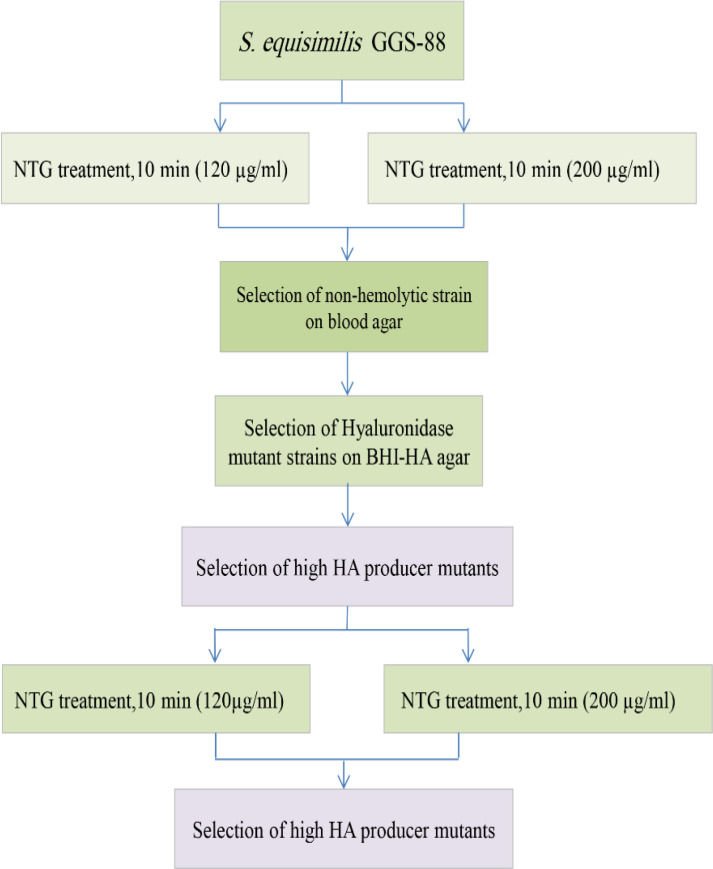
Procedure for selection of the GGS mutant strains

The schematic diagram of mutation and selection of GGS mutants is shown in Figure 1. For the selection of GGS mutants, a single colony of Gw was inoculated into 5 ml of BHI broth and incubated at 37 °C till the optical density (at 600 nm) reached 1.0. Fresh grown cells were collected by centrifugation at 10,000 ×g/5 min and washed with 0.05 M of phosphate buffer (pH 7.0). The bacterial pellet was resuspended in 2 ml of phosphate buffer containing 120 or 200 µg/ml of NTG and incubated at 37 °C for 10 min. Different incubation times (5, 10, 15, and 30 min) and NTG concentrations (100, 120, 200, 250, and 300 µg/ml) were applied, and the surviving strains were screened based on the mortality rate, morphology, and highly mucoid phenotype. The mutant bacteria were harvested and washed twice by phosphate buffer solution. Then the mutants were incubated in a 5-ml fresh BHI broth at 37 °C for 4 h. Subsequently, the bacteria were cultured on blood agar to select nonhemolytic strains^[^^16^^]^. The secondary screening was performed to identify hyaluronidase-negative mutants as explained before^[^^17^^]^. In brief, nonhemolytic colonies were cultured on BHI agar containing 1% HA and incubated at 37 °C for 18 h. Then cetylpyridinium chloride solution (10 % w/v) was sprayed on a plate and incubated at 37 °C for another 16 h. Finally, the nontransparent colonies were selected as hyaluronidase-negative mutants^[^^17^^]^.

HA production, optimization, and purification

The Gw and *S. zooepidemicus* (as a control strain without undergoing mutation) were incubated in BHI broth at 37 °C for 24 h. The pellets were then inoculated into the production medium (1.5 mg/ml of Na_2_HPO_4_.12H_2_O, 1.5 mg/ml of KH_2_PO_4_, 0.5 mg/ml of MgSO_4_.7H_2_O, 10 mg/ml of yeast extract, and 20 mg/ml of glucose) and incubated at different cultivation periods (4, 8, and 18 h) at 37 °C/220 rpm. The components of the production medium were justified for HA production according to previous studies^[^^13^^,^^17^^,^^18^^]^. The optimum pH of production reported ranging from 6.9 to 7.4 for GCS^[^^17^^,^^19^^,^^20^^]^, whereas, in our study, a broader pH range (4.5, 5.0, 5.5, and 7.0) was investigated in comparison to *S. zooepidemicus*. In addition, the HA concentration at different cultivation times (4, 8, and 18 h) was assessed. All experiments were performed in triplicate, and differences were determined by statistical analysis. For HA purification, an equal volume of 0.1% (w/v) of sodium dodecyl sulfate solution was added to the samples, and the mixtures were shaken gently for 10 min to liberate the capsular HA. Thereafter, the HA was precipitated by adding four volumes of ethanol and centrifugation at 10,000 ×g/15 min. The precipitated HA was resuspended in one volume of 1.5 M of NaCl and three volumes of ethanol and subsequently centrifuged at 10,000 g/15 min. Finally, the last sediment was resuspended in 200 µl of purified water for further analysis^[^^21^^]^.

Determination of HA concentration 

The standard curve of GlcUA (Merck, USA) was used to determine HA concentration in carbazole colorimetric assay. The sensitivity of the carbazole method has been reported approximately 1 µg for GlcUA^[^^22^^]^. A serial dilution of GlcUA at the concentrations of 400, 300, 200, 150, 100, 80, 64, 32, 16, and 8 µg/ml and diluted samples of 1:8 and 1:16 (50 µl) was placed in a 96 well microplate, and 200 µl of sodium tetraborate (25 mM in sulfuric acid) was added to each well. Then the plate was heated at 80 ºC for 20 min. After cooling at room temperature for 25 min, carbazole (50 µl; 0.125% in absolute ethanol) was added to the wells, and upon secondheating and cooling round, the plate was read in a microplate reader (Metertech, Taiwan) at a wavelength of 550 nm^[^^22^^,^^23^^]^. All experiments were performed in triplicates, and the coefficient of variation percentage less than 5% was considered the acceptance criteria. Following determination of HA titer, two higher producer strains (Gm1-120-21 and Gm1-200-10) among 34 mutant strains were selected for the second round of mutation. The nomination of mutants was based on the following criteria; (1) the first or second round of mutation (Gm1 or Gm2), (2) the used NTG concentration (120 or 200 μg/ml), and (3) the clone number. For instance, Gm2-120-10 stands for the 10^th^ clone of the second round of mutation at 120 μg/ml of NTG.

**Fig. 2:**
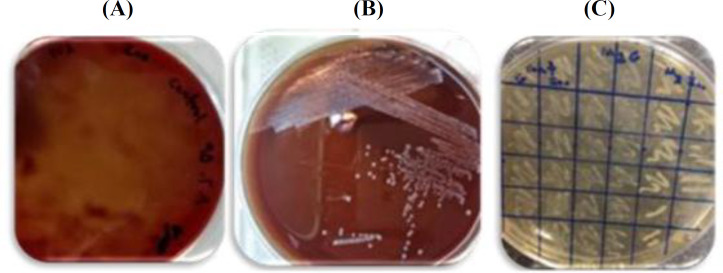
Morphology of the wild type and the mutant strains of Streptococcus. (A) The wild type of *S. zooepidemicus *with hemolytic activity; (B) the mucoid colony of a mutant strain (Gm1-120-2) without hemolytic activity; (C) Screening of hyaluronidase-negative strains on BHI agar containing 1% HA. Colonies in the right columns with non-transparent and opaque appearance show the hyaluronidase-negative feature in comparison to transparent appearance of Gw and *S. zooepidemicus* control strains at the left and central columns, respectively

Determination of Mw of HA by agarose gel electrophoresis

For Mw determination of HA products, 10 µg of the purified HA was loaded on 0.5 % agarose gel. The electrophoresis was performed in Tris-base, acetic acid and EDTA buffer (pH 8.3) at 60 V for 60 min in the presence of GeneRuler™ 1-kb DNA Ladder (GeneAll Co., South Korea). The gel was then soaked in 30% ethanol for 60 min and stained with Stains-All (Sigma; 0.1 mg/mL in 30 % ethanol) in darkness for at least 8 h^[^^24^^,^^25^^]^ .

Stability of HA production by the selected mutants 

Within 10 passages, the morphology consistency and HA production in high producers were assessed using both blood agar plate and carbazole assays.

Statistical analysis

The differences of HA titers in different medium conditions were analyzed by ANOVA method, at 5% probability level (*p* < 0.05). All statistical analyses were carried out using SPSS version 22.0 (SPSS, Inc., Chicago, IL).

## RESULTS

Selection of nonhemolytic and hyaluronidase-negative mutants 

Among 10 GCS/GGS strains, GGS-88 was selected due to the relatively high production of HA (579.7 µg/ml)^[^^13^^]^. NTG mutagenesis at the used concentrations (120 and 200 µg/ml) led to mutant strains with the following criteria: (1) low mortality rate, (2) negative streptolysin morphology, and (3) a highly mucoid phenotype. Mutated clones without hemolysis zone on blood agar plate were identified as nonhemolytic strains harboring inactivated streptolysin in comparison to beta-hemolytic Gw and *S. zooepidemicus* (Fig. 2). The hyaluronidase-negative mutants were also identified as nontransparent and opaque appearance colonies in the presence of HA (1%) after cetylpyridinium chloride treatment (Fig. 2).

**HA production, optimization, and determination**

**Fig. 3:**
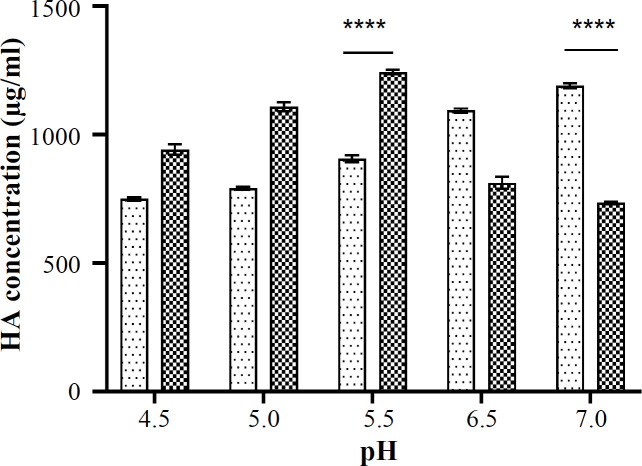
Effect of pH of culture medium on HA titer (941 ± 19.6, 1108 ± 17.0, 1241 ± 2.1, 812 ± 23.1 and 734 ± 5.5 µg/ml) for Gw (wild type of *S. equisimilis* GGS) and *S. zooepidemicus* (750 ± 6.6, 791 ± 6.4, 905 ±13.8, 1094 ± 7.9 and 1181 ± 5.4 µg/ml) at the pH range of 4.5- 7.0. ^****^ shows significant increase of HA titer

Evaluating the pH of production medium as well as cultivation time on HA titer showed that at lower pH, the Gw strain produced a higher titer of HA in a shorter time rather than *S. zooepidemicus*. The HA titer produced by Gw was 1241 ± 2.1, 1214 ± 3.2, and 1080 ± 3.5 μg/ml after 4, 8, and 18 hours, respectively. Statistical analysis indicated no significant difference between HA titers after 4 and 8 hours of cultivation time. The declining trend in HA titer for Gw strain might be related to the degradation of HA by secreted hyaluronidase and also to the decreasing carbon and nitrogen sources^[^^26^^]^. As shown in Figure 3, the HA titer was significantly high for *S. zooepidemicus* at pH 7.0, similar to a previous report on HA production by these strains^[^^12^^]^. The highest HA titer for Gw was obtained at pH 5.5 (*p* < 0.05) and 4 hours of cultivation, which was higher than that of other *S. zooepidemicus* strains (1241 ± 2.1 µg/ml versus 1094 ± 7.9 µg/ml)^[^^17^^,^^19^^,^^20^^]^. The optimum pH and cultivation time was applied in the rest of the study. 

HA production by mutant strains 

The Glucoronic acid standard curve was used to determine the HA titer. The results of the first round of NTG mutation are summarized in Table 1. In the first round, a wide range of HA titers was observed among colonies (a range of 851-1881 for 120 μg/ml NTG and a range of 911-1696 μg/ml for 200 μg/ml of NTG). Among mutant strains, Gm1-120-5 (1536 ± 7.6 μg/ml) and Gm1-120-21 (1881 ± 5.2 μg/ml) clones exhibited 24% and 52% increase in HA titer, respectively, at 120 μg/ml of NTG. At 200 μg/ml of NTG, Gm1-200-10 (1696 ± 2.8 µg/ml) and Gm1-200-16 (1470 ± 9.3 μg/ml) clones showed a 37% and 18% increment in the HA titer, respectively. Based on these results, Gm 1-120-21 and Gm 1-200-10 clones were selected as the high producers for the second round of mutation. Similar to the first round, the second round also led to colonies with a high variation in the HA titer. Five nonhemolytic and hyaluronidase-negative mucoid colonies were selected at the end of the second round of mutation. The HA titer among five mutants ranged from 1115 ± 4.2 μg/ml to 2856 ± 4.2 μg/ml (Table 2). Most of the strains showed a noticeable increase in the HA titer. Gm2-120-21-3, Gm2-120-21-4, Gm2-120-21-5, Gm2-200-10-4, and Gm2-200-10-5 clones exhibited 31.3%, 45.5%, 12.2%, 16.1%, and 25.6% increase in HA production, respectively, compared to the first generation.

**Results of the agarose gel electrophoresis**

**Fig. 4.**
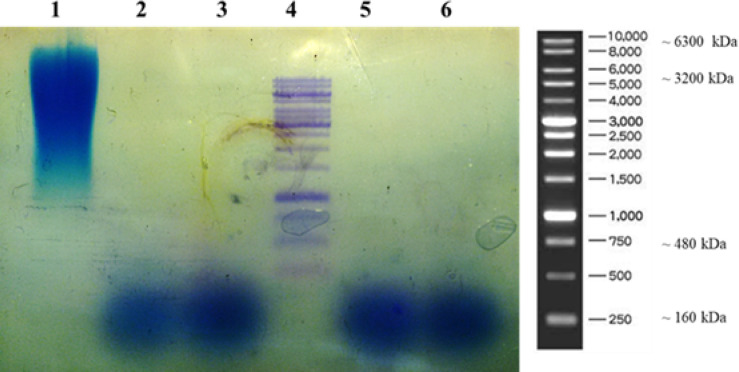
Electrophoresis of HA on 0.5% agarose. Lane 1, a polydisperse HA produced by *S. zooepidemicus*; lane 2, HA produced by GGS wild type; lane 3, HA produced by Gm2-200-10-5; lane 4, 1-Kb DNA ladder; lane 5, HA produced by Gm2-120-21-4;  lane 6: HA produced by Gm2-120-21-3. All wells were loaded with the same HA content (100 µg)

The findings of agarose gel electrophoresis demonstrated that HA produced by Gw and its mutant strains is low polydisperse with low-Mw (160 kDa), while *S. zooepidemicus* could produce a polydisperse polymer ranging from 3,200-10,000 kDa (Fig. 4).

**Table 1:** The HA titer produced by the mutants at the first round of chemical mutagenesis

**HA titer** ^*^ **(µg/ml)**	**Mutant strains** **(** **NTG 200 µg/ml)**	**No.**	**HA titer** ^*^ **(µg/ml)**	**Mutant strains** **(NTG 120 ** **µg/ml)**	**No.**
1352 ± 11.0	Gm1-200-2	1	1119 ± 6.2	Gm1-120-1	1
928 ± 6.6	Gm1-200-4	2	1027 ± 4.2	Gm1-120-2	2
1261 ± 5.2	Gm1-200-5	3	1107 ± 9.1	Gm1-120-4	3
891 ± 5.9	Gm1-200-9	4	1536 ± 7.6	Gm1-120-5	4
1696 ± 2.8	Gm1-200-10	5	1247± 2.4	Gm1-120-6	5
1200 ± 10	Gm1-200-14	6	1102 ± 6.6	Gm1-120-8	6
1470 ± 9.3	Gm1-200-16	7	1241 ± 3.8	Gm1-120-9	7
911 ± 3.6	Gm1-200-17	8	1137 ± 8.7	Gm1-120-11	8
1173 ± 9.0	Gm1-200-18	9	1261± 5.0	Gm1-120-13	9
927 ± 5.6	Gm1-200-20	10	851 ± 5.2	Gm1-120-15	10
1428 ± 3.1	Gm1-200-23	11	1357 ± 7.1	Gm1-120-16	11
1516 ± 2.5	Gm1-200-26	12	1881 ± 5.2	Gm1-120-21	12
1384 ± 2.1	Gm1-200-27	13	1219 ± 8.3	Gm1-120-24	13
983 ± 6.5	Gm1-200-30	14	867 ± 9.3	Gm1-120-27	14
1115 ± 4.2	Gm1-200-32	15	1142 ± 6.7	Gm1-120-29	15
1089 ± 4.7	Gm1-200-33	16	1130 ± 2.8	Gm1-120-30	16
958 ± 5.5	Gm1-200-37	17	-	-	-
1330 ± 7.8	Gm1-200-38	18	-	-	-

^*^Standard deviation was calculated from three individual experiment.

**Table 2:** The HA titer produced by the mutants at the second round of chemical mutagenesis

HA-titer^*^ (µg/ml)	Mutant strains (NTG 200 µg/ml)	No.	HA-titer^*^ (µg/ml)	Mutant strains (NTG 120 µg/ml)	No.
1115 ± 8.3	Gm2-200-10-1	1	1927 ± 6.2	Gm2-120-21-1	**1**
1579 ± 3.1	Gm2-200-10-2	2	1756 ± 7.2	Gm2-120-21-2	**2**
1792 ± 9.0	Gm2-200-10-3	3	2470 ± 8.1	Gm2-120-21-3	**3**
1969 ± 2.1	Gm2-200-10-4	4	2856 ± 4.2	Gm2-120-21-4	**4**
2231± 6.1	Gm2-200-10-5	5	2113 ± 9.4	Gm2-120-21-5	**5**

^*^Standard deviation was calculated from three individual experiment.

Stability of the selected mutant 

Stability assessment of the selected mutants, including Gm2-120-21-3 and Gm2-120-21-4 clones, revealed that both morphology and productivity of HA were stable. Monitoring from the 1^st^ to 10^th^ generation did not show any noticeable variation in the HA titer. The change in HA titer was from 2470 to 2529 µg/ml for the Gm2-120-21-3 and from 2856 to 2763 µg/ml for the Gm2-120-21-4 clones.

## DISCUSSION

Strain development, optimization of cultivation and purification are the most important steps for production of biomaterials by microorganisms^[^^7^^,^^10^^]^. In this study, the optimum condition was assayed for HA production by using Gw and *S. zooepidemicus* at various pH values and incubation times. 

The results showed that the high HA titer was obtained at pH 5.5 and 4 hours of cultivation for Gw, which was different from *S. zooepidemicus* reported in previous studies (a pH range of 6.9-7.4 and 16-24 hours of cultivation)^[^^17^^,^^19^^,^^20^^]^. This optimum condition confirms the advantage of using GGS for HA production due to the shorter time of cultivation. In addition, higher productivity at lower pH provides the possibility for using acidic renewable resources such as agricultural derivatives, cheap crude materials, and wastes from other industrial processes that leads to a reduced cost^[^^10^^]^. In the current study, we developed high-producing GGS mutants for HA production by random chemical mutagenesis. The NTG mutagenesis, same as other physical or chemical random mutation methods, led to the variable results^[^^9^^,^^17^^,^^27^^]^. Although all mutated clones were nonhemolytic and hyaluronidase-negative in phenotype, a decline in HA production was observed in several clones. For instance, in the first round of mutation at NTG concentration of 120 µg/ml, Gm1-120-15 and Gm1-120-17 clones showed 851 and 867 µg/ml of HA titer with a 31.4% and 30.1% decrease in HA production, respectively. The Gm1-200-20 and Gm1-200-9 mutant strains indicated a 20.8% to 28.2% reduction in HA titer, respectively, at NTG concentration of 200 µg/ml. However, after the second round of random mutation, multiple clones, including Gm1-120-21 (51.5 %) and Gm1-200-10 (36.7 %), displayed a noticeable increase in HA production. Similar to the first round, the second round of mutation also resulted in different outputs. Gm2-120-21-3, Gm2-120-21-4, and Gm2-120-21-5 clones showed a 31.3%, 45.5%, and 12.2% increment more than their parent (Gm1-120-21) and 2-, 2.3-, and 1.7-folds greater than Gw strain as the original strain, respectively. Accordingly, Gm2-200-10-4 and Gm2-200-10-5 clones exhibited a 16.1% and 25.6% increase in HA titer rather than their parent (Gm1-200-10) and 1.6- and 1.7-folds greater than Gw strain, respectively. The HA titer comparison between the first and second rounds of mutation indicated that higher concentrations of NTG do not necessarily lead to higher-producing strains. The variation in the HA production was expected and highlighted the role of genes that mediate HA biosynthesis. This phenomenon elucidated that the involved genes may be affected negatively or positively by random mutations. 

The HA biosynthesis pathway in *Streptococcal* strains is similar in requirement of precursor monosaccharides (GlcUA-UDP and GluNAc-UDP) and HA synthase enzyme^[^^8^^]^. The* has* locus containing *has*B gene encodes UDP-glucose dehydrogenase, which converts UDP-glucose to UDP-glucuronic acid, and *has*C gene encodes UDP-glucose pyrophosphorylase (glucose-1-phosphate uridylyl transferase), which converts glucose-1-phosphate to UDP-glucose^[^^7^^]^. This locus is highly conserved among *Streptococcal *strains^[^^8^^]^. Using site-directed mutagenesis, *S. zooepidemicus* mutant strains harboring a single point mutation in the upstream region of *has*A promoter showed an increase (5-16%) and a decrease (41%) in the HA titer^[^^9^^]^, indicating the importance of the genetic role in HA productivity. Therefore, the genetic analysis of those strains with significant increase in HA production after random mutation would be helpful in identification of the hot spots within the HA operon (*has* A, *has* B, and *has* C) for the rational design of high-producing GGS strains. 

Several studies have been conducted to develop mutant strains for HA production by chemical, physical, and combination methods such as UV radiation, Co-γ, 5-BrU, and NTG. In the mutagenesis step, the microorganisms are exposed to the mutagen or the ray at different times and concentrations/ intensities^[^^12^^,^^17^^,^^27^^]^. However, the development of reliable high-throughput screening methods would be necessary for rapid mutant selection. In addition, optimization of downstream steps, including culture conditions, extraction, and purification process, enhances HA productivity^[^^10^^,^^19^^,^^28^^]^. It has been represented that the HA production by *S. equisimilis* and *S. zooepidemicus* mutant strains at the optimized fermentation conditions could increase the HA titer from 0.3 mg/ml and 0.6 mg/ml to 4.12 mg/ml and 6.7 mg/ml, respectively^[^^16^^,^^29^^,^^30^^]^. Besides, studies have proved the significant effect of medium components and cultivation conditions on HA productivity^[^^21^^,^^31^^,^^32^^]^. Regarding the high HA titers obtained from Gm2-200-10-5, Gm2-120-21-3, and Gm2-120-21-4 clones, it can be concluded that medium production optimization can remarkably improve the HA titer. The stability study on high producer strains such as Gm2-120-21-3 and Gm2-120-21-4 confirmed a consistent production within 10 generations, which make them suitable candidates for industrial production of HA. The gel electrophoresis revealed that the mutant strains can produce a relatively low polydisperse polymer. The HA with a Mw ranging from 80 to 800 kDa is considered low Mw HA, which is biologically active^[^^33^^,^^34^^]^. Low Mw of HAs mediates leukocyte, fibroblast, and endothelial migration and activation which is used in wound healing and angiogenesis^[^^6^^,^^35^^,^^36^^]^. These HAs are mostly obtained by enzymatic digestion or ultrasonic degradation of high Mw HA^[^^10^^]^. However, in the current study, the agarose gel electrophoresis showed that the *S. zooepidemicus* produces the polydisperse high Mw HA, as expected to be similar to previous reports^[^^12^^,^^28^^]^ (Fig. 4), while the production of low polydisperse and low Mw HA by wild and mutant GGS strains would be a noticeable advantage. 

Overall, we introduce the novel GGS wild type and the mutant ones with the ability of HA production at lower pH and shorter time rather than the usual conditions for *Streptococcal* fermentation. The mutant strains could produce a high titer of HA than the wild type, which can be considered as suitable producers for large-scale production. More importantly, the low Mw and low polydisperse properties of the produced HAs suggest their potential applications in biology and medicine.

## DECLARATIONS

### Acknowledgments

All authors would like to thank Nano-Biotechnology department of Pasteur Institute of Iran.

### Ethical statement

Not applicable.

### Data availability

The raw data supporting the conclusions of this article are available from the corresponding author on reasonable request.

### Author contributions

BJ: conducted the experiments and wrote the drafts; MK: received the grant, supervised the project, and revised and finalized the manuscript; RAC: revised and finalized the manuscript; SMA: contributed new reagents or analytical tools; SAH: conducted the experiments. All authors read and approved the manuscript

### Conflict of interest

None declared.

### Funding/support

This research was financially supported by the Pasteur Institute of Iran (Tehran), research grant no. 871.
